# Altered sleep spindles and slow waves during space shuttle missions

**DOI:** 10.1038/s41526-021-00177-1

**Published:** 2021-11-18

**Authors:** Dominik P. Koller, Vida Kasanin, Erin E. Flynn-Evans, Jason P. Sullivan, Derk-Jan Dijk, Charles A. Czeisler, Laura K. Barger

**Affiliations:** 1 Advanced Concepts Team, European Space Agency, ESTEC, Noordwijk, The Netherlands; 2 Fatigue Countermeasures Laboratory, Human Systems Integration Division, Exploration Technology Directorate, NASA Ames Research Center, Moffett Field, CA USA; 3 Division of Sleep and Circadian Disorders, Department of Medicine, Brigham and Women’s Hospital, Boston, MA USA; 4 Surrey Sleep Research Centre, Faculty of Health and Medical Sciences, University of Surrey, Guildford, UK; 5 UK Dementia Research Institute Care Research and Technology Centre, Imperial College London and the University of Surrey, Guildford, UK; 6 Division of Sleep Medicine, Harvard Medical School, Boston, MA USA

**Keywords:** Neuroscience, Human behaviour, Occupational health, Fatigue, Physiology

## Abstract

Sleep deficiencies and associated performance decrements are common among astronauts during spaceflight missions. Previously, sleep in space was analyzed with a focus on global measures while the intricate structure of sleep oscillations remains largely unexplored. This study extends previous findings by analyzing how spaceflight affects characteristics of sleep spindles and slow waves, two sleep oscillations associated with sleep quality and quantity, in four astronauts before, during and after two Space Shuttle missions. Analysis of these oscillations revealed significantly increased fast spindle density, elevated slow spindle frequency, and decreased slow wave amplitude in space compared to on Earth. These results reflect sleep characteristics during spaceflight on a finer electrophysiological scale and provide an opportunity for further research on sleep in space.

## Introduction

Sleep deficiencies among astronauts have been reported across spaceflight missions for decades^1^. Most studies using self-reports based on subjective ratings and objective measures through actigraphy and electroencephalography (EEG; scalp-attached electrodes monitoring brain activity) have indicated reduced sleep quality, sleep duration, sleep efficiency, sleep period time (SPT), and physical restedness, as well as increased sleep latency^2–7^. Only a few self-reported sleep quality measures seem to suggest otherwise^1^. The majority of astronauts resort to the use of sleep-promoting drugs^2^. In addition, reported sleep deficiencies seem to persist despite improvements in environmental sleeping conditions^7^, raising questions of whether microgravity itself may play a role in physiologically altering sleep.

Given that sleep deficiencies are associated with fatigue and compromised cognitive functioning^8^, failing to identify the underlying causes of sleep deficiencies in space could result in an increased risk of human errors and accidents during long-term spaceflight missions. Some evidence from spaceflight studies supports this concern, indicating inflight increases in fatigue^9^ and decrements in performance on certain cognitive tasks, such as memory-search^10^, tracking abilities and time-sharing efficiency^11,12^, dual-task performance^13^, and word recall^3^.

Previous studies on sleep in space using EEG have investigated overall differences in sleep architecture, showing reduced total sleep duration^6^, decreased time spent in slow wave sleep^3,6^, and redistribution of slow wave sleep^5,14^. However, EEG data were never analyzed for changes of individual neural oscillations during sleep, such as sleep spindles and slow waves, which characterize certain sleep stages and are known to be related to changes in sleep quality with ageing^15^. To further elucidate the underlying causes of sleep deficiencies in space the present study investigates sleep spindle and slow wave responses to spaceflight with a focus on discrete oscillations.

Sleep spindles are 0.5–2 s electrical bursts with a frequency between 9 and 15 Hz generated in the reticular nucleus of the thalamus and are a characteristic of stage 2 non-REM sleep (N2) but also occur in stage 3 non-REM sleep (N3)^16,17^. Sleep spindles are postulated to suppress sensory processing during sleep, which may be essential to sustain sleep, and could as such indirectly contribute to sleep quality^18–22^. Furthermore, pre-sleep declarative memory training, as well as overnight declarative memory retention, are correlated with increased sleep spindle density, inferring a key role of spindles in declarative memory consolidation^23,24^. Sleep spindles occurring during N3, and the coupling between slow oscillations and spindles might play a particularly important role in memory consolidation^25,26^. More recent studies suggest the existence of two distinct types of sleep spindles: slow spindles typically oscillating between 9 and 12 Hz and fast spindles with frequencies between 12 and 15 Hz^27^. Both spindle types show distinct haemodynamic responses^28^, different topographic distributions^29^, occur at different phases of slow waves^27^, and exhibit phenotypic and genetic heterogeneity^17^. These findings indicate distinct generating mechanisms and hint at disparate functional roles. Increases in fast spindle density, spectral power, and duration have been associated with motor sequence learning, while no such relation was found for slow spindles^30–34^. Furthermore, the performance in visuomotor learning tasks was found to be correlated with fast spindle amplitude and duration^35,36^. Slow spindle density and activity on the other hand were found to be associated with overnight word-pair retention^37^. In addition, both types of spindles have been related to general cognitive and learning abilities^38^.

Slow waves (SWs) are characteristic of N3, i.e., “deep sleep” or “slow-wave sleep” (SWS). SWs have frequencies of 0.5−3.5 Hz with peak-to-peak amplitudes of >75 μV^39^. The evidence so far suggests the importance of SWs in memory consolidation^40^, cognitive performance^41,42^, and sleep-dependent synaptic renormalization^43,44^. In addition, SWs make up the sleep stage most resilient against environmental interference^45–47^. Furthermore, reduced SW band-power, which positively correlates with SW amplitude^48^, was found to predict lighter sleep^49^, a shortened sleep duration^50^, reduced sleep efficiency^51,52^, and increased number of nocturnal awakenings^47^.

Considering the literature described above, reduced sleep duration and fragmentation during spaceflight could be associated with reduced sleep spindle density, therefore, we hypothesized that sleep spindle density during sleep stages N2 and N3 would be reduced inflight compared to preflight and postflight. We also aimed to characterize sleep spindle spectral band-power, amplitude, duration, and frequency. Moreover, reduced sleep duration and fragmentation could also be related to decreased SW amplitudes. For this reason, we further hypothesized that SW amplitudes during sleep stage N3 would be reduced inflight compared to preflight and postflight. Additionally, we aimed to characterize slow wave density, spectral band-power, duration, and slope. To test these hypotheses, EEG sleep data that were collected before, during, and after two Space Shuttle missions were analyzed^3^.

## Results

### Sleep statistics

In the present study, we analyzed full night sleep EEG data that were recorded from four astronauts (*n* = 4) before, during, and after two Space Shuttle missions. Notably, the astronauts were given melatonin or a placebo during some of the nights in the investigated experiments^3^. The effects of melatonin were not of primary interest to our study, therefore, we controlled for the administration of the hypnotic drug with our statistical models (Linear mixed-effects models; LMMs). To understand the differences in global sleep measures of our cohort of astronauts compared to previous studies, we investigated their SPT and total sleep time (TST), as well as changes in sleep stages between the three experimental conditions (pre-, in-, and postflight).

SPT was 41.98 ± 7.89 min less inflight compared to preflight and 34.30 ± 9.86 min less compared to postflight (Condition: *F*(2,11.14) = 15.23, *p* < 0.01; Table 1). SPT was not affected by Melatonin (*F*(1,12.46) = 1.47, *p* = 0.25).

**Table 1:** Summary of post hoc estimated marginal means contrasts following a significant effect in the linear mixed-effects models.

Contrast	Effect size	SE	df	*t*	*p*	CI_95%_ lower	CI_95%_ higher
Sleep period time (min)							
IN-POST	−34.301	9.863	11.136	−3.478	0.013*	−60.892	−7.710
IN-PRE	−41.976	7.890	11.136	−5.320	0.001*	−63.249	−20.703
POST-PRE	−7.676	9.863	11.136	−0.778	0.723	−34.267	18.915
Total sleep time (min)							
IN-POST	−32.044	19.950	11.042	−1.606	0.284	−85.897	21.809
IN-PRE	−48.161	15.960	11.042	−3.018	0.029*	−91.243	−5.079
POST-PRE	−16.117	19.950	11.042	−0.808	0.706	−69.969	37.736
Fast spindle density (1/min)							
IN-POST	0.351	0.660	26.000	0.532	0.856	−1.288	1.991
IN-PRE	1.757	0.528	26.000	3.329	0.007*	0.446	3.069
POST-PRE	1.406	0.660	26.000	2.131	0.103	−0.233	3.046
Slow spindle frequency (Hz)							
IN-POST	0.145	0.038	26.000	3.834	0.002*	0.051	0.239
IN-PRE	0.170	0.030	26.000	5.625	<0.001*	0.095	0.245
POST-PRE	0.025	0.038	26.000	0.666	0.785	−0.069	0.119
Overall spindle frequency (Hz)							
IN-POST	0.138	0.072	26.000	1.929	0.151	−0.040	0.316
IN-PRE	0.266	0.057	26.000	4.652	<0.001*	0.124	0.409
POST-PRE	0.128	0.072	26.000	1.793	0.192	−0.049	0.306
Slow wave amplitude (µV)							
IN-POST	−1.985	0.877	11.000	−2.262	0.104	−4.355	0.385
IN-PRE	−2.530	0.702	11.000	−3.604	0.011*	−4.426	−0.634
POST-PRE	−0.545	0.877	11.000	−0.621	0.812	−2.915	1.825

Presented are the unstandardized effect sizes (effect size), standard error of the estimates (SE), degrees of freedom (df), *t*-values (*t*), *p*-values (*p*), and the lower and higher 95%-confidence intervals (CI_95%_). Significant *p*-values of *p* < 0.05 are indicated by *.

TST was significantly reduced by 48.16 ± 15.96 min inflight compared to preflight (Condition: *F*(2,11.04) = 4.65, *p* = 0.03; Table 1). Melatonin did not significantly influence TST (*F*(1,11.98) = 0.08, *p* = 0.78).

In addition, the duration of sleep stages and WASO did not differ depending on Condition (*F*(2,71.14) = 0.90, *p* = 0.41) and was not affected by Melatonin (*F*(1,72.85) = 0.09, *p* = 0.77). Furthermore, there was no significant interaction of Condition with any of the sleep stages (*F*(8,71.14) = 0.63, *p* = 0.75). As expected, the duration of each sleep stage varied significantly between stages (*F*(4,71.14) = 68.66, *p* < 0.01). The reductions of TST and SPT are likely related to non-significant reductions of certain sleep stages and increases in WASO (see Supplementary Fig. 1).

### Fast, slow, and overall sleep spindle characteristics

**Fig. 1:**
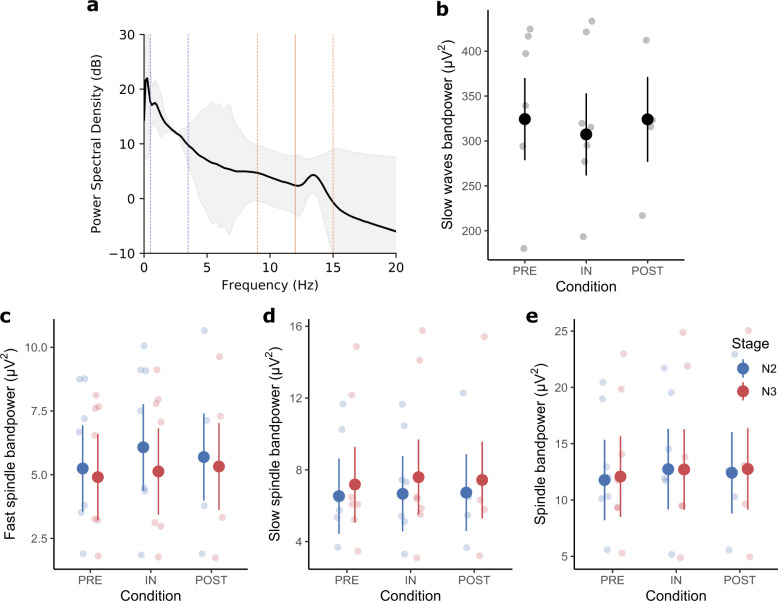
Power spectral density analyses in N2 and N3. **a** Power spectral density: The thick black line represents the average power across all channels, conditions, sessions, and subjects across N2 and N3, while the shaded gray area indicates the standard error. A solid orange line separates the a priori defined slow spindle (9–12 Hz) and fast spindle (12–15 Hz) ranges. Two dashed orange lines show the lower-bound (9 Hz) and upper-bound (15 Hz) of the total spindle frequency band. A clear PSD increase can be observed for frequencies of around 13.5 Hz. Two purple dashed lines indicate the a priori defined slow wave band (0.5–3.5 Hz). **b** Slow wave band-power: was calculated by integrating over the slow wave band in (**a**) and finally used in a linear mixed-effects model to evaluate statistical differences between conditions. **c** Fast spindle band-power: was calculated similar to (**b**). **d** Slow spindle band-power: was calculated similar to (**b**). **e** Overall spindle band-power: was calculated similar to (**b**). For **b**, **c**, **d**, and **e** the large dots represent the estimated marginal means (± standard error) of each condition separated for stages N2 and N3 in the spindle analyses. The smaller dots in the background are individual data points.

Next, we detected fast (12–15 Hz) and slow (9–12 Hz, Fig. 1a) sleep spindles during N2 and N3 in the sleep EEG of the astronauts and analyzed their individual characteristics.

**Fig. 2:**
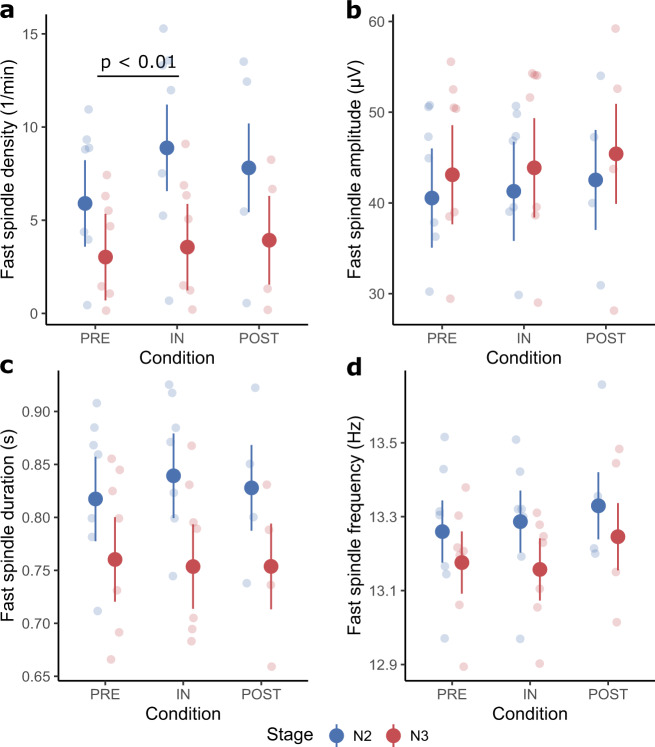
Fast spindle characteristics. Large dots represent the estimated marginal means (± standard error) of each condition separated by sleep stage. The smaller dots in the background are individual data points in the respective condition and sleep stage. Significant differences (except between stages) are indicated by a black bar with an annotation stating the *p*-value. **a** Shows a significant difference of fast spindle densities between preflight and inflight. **b** Shows the fast spindle amplitude. **c** Shows the fast spindle duration. **d** Shows the fast spindle frequency.

Fast spindle densities significantly differed between the conditions (*F*(2,26) = 5.92, *p* < 0.01; Fig. 2a) and stages (*F*(1,26) = 69.98, *p* < 0.01; Supplementary Table 1). Post hoc tests revealed that fast spindle density significantly increased by 1.76 ± 0.53 spindles/min inflight compared to preflight (Table 1). The interaction Condition × Stage (*F*(2,26) = 2.71, *p* = 0.09) and Melatonin were not significant (*F*(1,26.07) = 0.43, *p* = 0.52).

Fast spindle amplitude (Fig. 2b) was not affected by Condition (*F*(2,26) = 3.05, *p* = 0.06), the Condition × Stage interaction (*F*(2,26) = 0.02, *p* < 0.98) nor Melatonin (*F*(1,26.02) = 1.92, *p* = 0.18). Stage was the only factor that significantly modulated fast spindle amplitude (*F*(1,26) = 17.70, *p* < 0.01; Supplementary Table 2).

Spectral band-power in the fast spindle range (Fig. 1c) only deviated significantly between sleep stages (*F*(1,26) = 7.91, *p* < 0.01; Supplementary Table 3) all other factors were non-significant (Condition: *F*(2,26) = 3.24, *p* = 0.06; Condition × Stage: *F*(2,26) = 1.18, *p* = 0.32; Melatonin: *F*(1,26.02) = 2.31, *p* = 0.14).

Fast spindle duration (Fig. 2c) was only affected by Stage (*F*(1,26) = 141.61, *p* < 0.01; Supplementary Table 4), while all other factors were non-significant (Condition: *F*(2,26) = 0.68, *p* = 0.52; Condition × Stage: *F*(2,26) = 2.32, *p* = 0.12; Melatonin: *F*(1,26.04) = 0.00, *p* = 0.98).

Sleep stage influenced the frequency (*F*(1,26) = 9.90, *p* < 0.01; Fig. 2d and Supplementary Table 5) of fast spindles across all conditions, however, all other factors had no significant effect (Condition: *F*(2,26.00) = 1.48, *p* = 0.25; Condition × Stage: *F*(2,26.00) = 0.27; *p* = 0.77, Melatonin: *F*(1,26.25) = 2.25, *p* = 0.15).

**Fig. 3:**
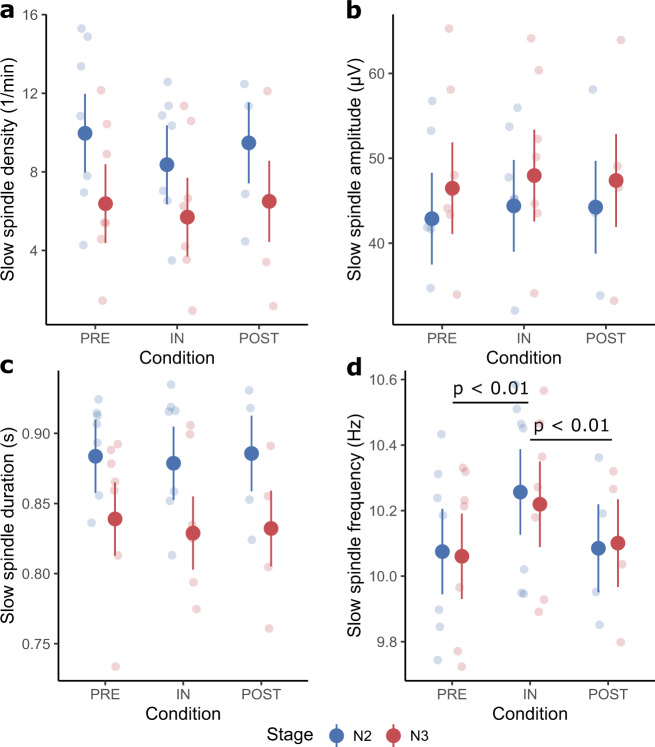
Slow spindle characteristics. Large dots represent the estimated marginal means (± standard error) of each condition separated by sleep stage. The smaller dots in the background are individual data points in the respective condition and sleep stage. Significant differences (except between stages) are indicated by a black bar with an annotation stating the *p*-value. **a** Shows the slow spindle density. **b** Shows the slow spindle amplitude. **c** Shows the slow spindle duration. **d** Shows the slow spindle frequency with a significant difference between preflight and inflight as well as postflight and inflight.

Slow spindle density (Fig. 3a) did not significantly differ between conditions (*F*(2,26) = 3.13, *p* = 0.06), and was not affected by the Condition × Stage interaction (*F*(2,26) = 0.47, *p* = 0.63). Slow spindle density was significantly affected by Stage (*F*(1,26) = 50.20, *p* < 0.01; Supplementary Table 11) and Melatonin (*F*(1,26.08) = 7.36, *p* = 0.01; Supplementary Table 12).

For slow spindle amplitude only the factor Stage was significant (*F*(1,26) = 19.43, *p* < 0.01; Fig. 3b and Supplementary Table 13), while all other factors had no significant effect (Condition: *F*(2,26) = 1.63, *p* = 0.22, Condition × Stage: *F*(2,26) = 0.03, *p* = 0.97, Melatonin: *F*(1,26.03) = 3.22, *p* = 0.08).

Slow spindle band-power (Fig. 1d) was neither affected by Condition (*F*(2,26) = 0.22, *p* = 0.80), Stage (*F*(1,26) = 3.90, p = 0.06), Melatonin (*F*(1,26.05) = 1.24, *p* = 0.28) nor the Condition × Stage interaction (*F*(2,26) = 0.06, *p* = 0.94).

For slow spindle duration (Fig. 3c) only the factor Stage was significant (*F*(1,26) = 60.71, *p* < 0.01; Supplementary Table 14). All other factors did not significantly modulate the duration (Condition: *F*(2,26) = 0.60, *p* = 0.56, Condition × Stage: *F*(2,26) = 0.16, *p* = 0.85, Melatonin: *F*(1,26.10) = 1.35, *p* = 0.26)

Slow spindle frequency (Fig. 3d) was significantly affected by Condition (*F*(2,26) = 17.31, *p* < 0.01), while all other factors had no significant effect (Stage: *F*(1,26) = 0.19, *p* = 0.67; Melatonin: *F*(1,26.07) = 0.07, *p* = 0.80; Condition × Stage: *F*(2,26) = 0.28, *p* = 0.76). Post hoc results revealed a slow spindle shift towards higher frequencies by 0.17 ± 0.03 Hz inflight compared to preflight (Table 1). Upon return to Earth, the slow spindle frequency reverted to the preflight baseline (Table 1).

Additionally, we repeated the same analysis for sleep spindles detected across the entire spindle band from 9 to 15 Hz (Fig. 1a) to make our findings readily comparable to studies that focused on overall sleep spindles and did not distinguish fast and slow spindle types.

**Fig. 4:**
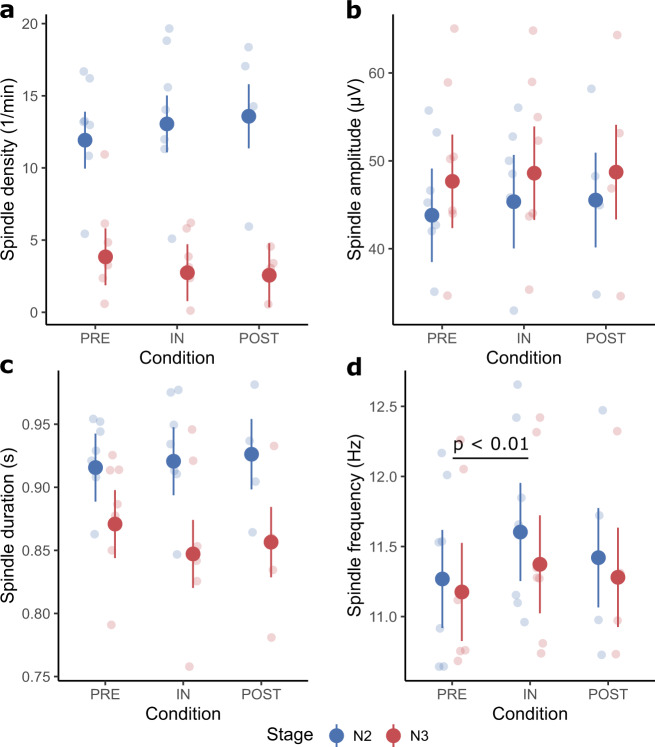
Overall spindle characteristics. Large dots represent the estimated marginal means (± standard error) of each condition separated by sleep stage. The smaller dots in the background are individual data points in the respective condition and sleep stage. Significant differences (except between stages) are indicated by a black bar with an annotation stating the *p*-value. **a** Shows the overall spindle density. **b** Shows the overall spindle amplitude. **c** Shows the overall spindle duration. **d** Shows the overall spindle frequency with a significant difference between preflight and inflight.

Overall sleep spindle density (Fig. 4a) did not significantly differ between conditions (*F*(2,26) = 0.01, *p* = 0.99) and was not affected by the Condition × Stage interaction (*F*(2,26) = 0.94, *p* = 0.40). Stage turned out to have a significant effect on sleep spindle density (*F*(1,26) = 109.91, *p* < 0.01; Supplementary Table 20) while Melatonin had no detectable influence (*F*(1,26.43) = 0.36, *p* = 0.55).

Sleep spindle amplitude (Fig. 4b) was not affected by Condition (*F*(2,26) = 1.38, *p* = 0.27) nor the Condition × Stage interaction (*F*(2,26) = 0.09, *p* = 0.92). The sleep stage significantly modulated the spindle amplitude (*F*(1,26) = 19.65, *p* < 0.01; Supplementary Table 21). The administration of melatonin also affected the spindle amplitude (*F*(1,26.03) = 5.13, *p* = 0.03; Supplementary Table 22).

Sleep spindle band-power (Fig. 1e) was not significantly affected by any of the factors (Condition: *F*(2,26) = 1.38, *p* = 0.27; Condition × Stage: *F*(2,26) = 0.07, *p* = 0.93; Stage: *F*(1,26) = 0.21, *p* = 0.65; Melatonin: *F*(1,26.03) = 2.59, *p* = 0.12).

Sleep spindle duration (Fig. 4c) was only affected by Stage (*F*(1,26) = 91.59, *p* < 0.01; Supplementary Table 23), while all other factors were non-significant (Condition: *F*(2,26) = 0.90, *p* = 0.42; Condition × Stage: *F*(2,26) = 2.25, *p* = 0.13; Melatonin: *F*(1,26.10) = 1.37, *p* = 0.25).

Condition significantly affected sleep spindle frequency (*F*(2,26) = 10.83, *p* < 0.01; Fig. 4d). Post hoc tests indicated that the spindle frequency increased by 0.27 ± 0.06 Hz inflight compared to preflight (Table 1). In addition, the factor Stage turned out to be significant (*F*(1,26) = 8.72, *p* < 0.01; Supplementary Table 24), while the remaining factors did not affect the frequency (Condition × Stage: *F*(2,26) = 0.73, *p* = 0.49; Melatonin: *F*(1,26.04) = 3.17, *p* = 0.09).

### Slow wave characteristics

Finally, we detected and analyzed individual slow waves during N3 in the sleep EEG of the astronauts within a frequency range from 0.5 to 3.5 Hz (Fig. 1a).

**Fig. 5:**
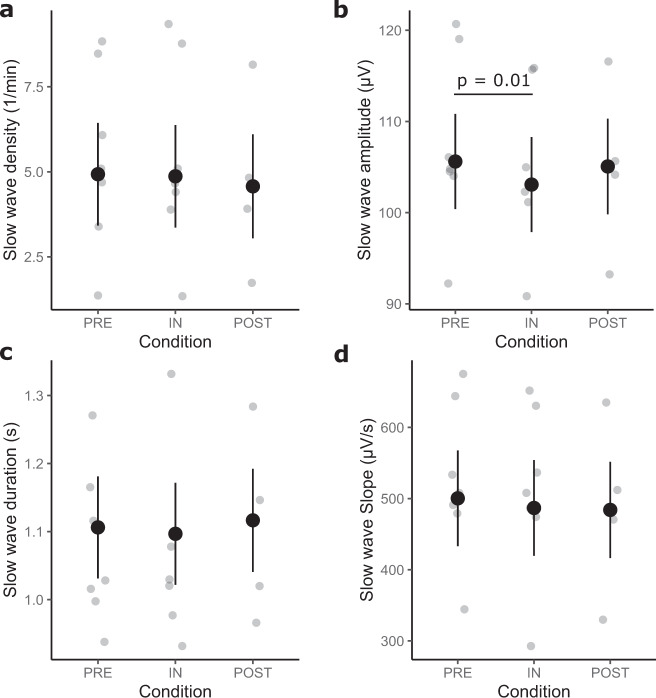
Slow wave characteristics. Large dots represent the estimated marginal means (± standard error) of each condition. The smaller dots in the background are individual data points. Significant differences are indicated by a black bar with an annotation stating the *p*-value. **a** Shows the slow wave density. **b** Shows the slow wave amplitude which significantly differed inflight compared to preflight. **c** Shows the slow wave duration. **d** Shows the slow wave slope.

SW density (Fig. 5a) did not significantly differ between levels of the factors included in the model (Condition: *F*(2,11) = 0.40, *p* = 0.68; Melatonin: *F*(1,11.03) = 0.23, *p* = 0.64).

In contrast, Condition affected SW amplitude (*F*(2,11) = 6.89, *p* = 0.01; Fig. 5b), which decreased by 2.53 ± 0.70 µV inflight compared to preflight (Table 1). Melatonin was non-significant (*F*(1,11.01) = 0.19, *p* = 0.67).

Neither SW duration (Condition: *F*(2,11) = 0.65, *p* = 0.54; Melatonin: *F*(1,11.02) = 2.77, *p* = 0.12; Fig. 5c) nor SW slope (Condition: *F*(2,11) = 1.70, *p* = 0.23; Melatonin: *F*(1,11.01) = 0.37, *p* = 0.55; Fig. 5d), nor SW band-power (Condition: *F*(2, 11) = 0.76, *p* = 0.49; Melatonin: *F*(1, 11.06) = 0.77, *p* = 0.40; Fig. 1b) were significantly affected by any of the factors.

## Discussion

Previous studies have mainly investigated overall sleep architecture and found reduced sleep time, reduced SW sleep and performance decrements during spaceflight missions^2,3,5,6,14,53^. However, these studies did not focus on the properties of the neural oscillations defining the sleep stages that make up astronauts’ sleep architecture. In the present study, we investigated individual sleep spindles and SWs that were detected in sleep recordings acquired before, during, and after Space Shuttle missions.

In the study by Dijk et al.^3^ actigraphy (movement-based measure of rest-activity cycles) estimated SPT was reduced by 34.8 min inflight compared to postflight. In the present study, a comparable reduction of SPT by 34.30 min was found inflight compared to postflight using an estimate based on the EEG recordings. In addition, SPT was found to be decreased by 41.98 min inflight compared to preflight. In contrast to Dijk et al.^3^ a significant decrease of EEG-derived TST by 48.16 min during inflight compared to preflight was found. It seems likely that the observed effects were mainly driven by different people in the cohort as there was incomplete overlap in subjects between the two studies. In addition, the variability controlled for by the employed statistical methods (Linear mixed-effects models) might have facilitated the detection of the SPT and TST changes. The observed reduction of SPT and TST is consistent with other EEG-based^6^ and actigraphy-based studies^2^ that showed a decrease in sleep duration.

Studies of sleep EEG in space have found a reduction of time spent in SWS^3,6^ and a redistribution of SWS from the first to the second sleep cycle^5,14^. Although Dijk et al.^3^, reported a decrease in SWS in the final third of the sleep episode, the present study found no difference in the duration of sleep stage N3 reflecting SWS across the night; however, peak-to-peak amplitude of discrete SWs was decreased inflight compared to preflight. Postflight SW amplitudes did not deviate significantly from inflight nor preflight but had a magnitude comparable to preflight SW amplitudes indicating an immediate renormalization after return to Earth. This is the first evidence that SW characteristics are affected during spaceflight. On Earth, lower SW amplitudes are known to correlate with reduced SW band-power^48^, which in turn is associated with lighter sleep^49^, a shortened sleep duration^50^, reduced sleep efficiency^51,52^ and increased number of nocturnal awakenings^47^; however, changes in SW band-power could not be detected in this study, possibly because these changes are pronounced in frontal electrodes^15^ and/or because of a low sample size. Yet the reduced peak-to-peak amplitude of SWs is particularly interesting given the shortened SPT and TST observed in this study and during human spaceflight in general. Notably, reduced SW amplitudes along with shortened sleep duration have been linked to aging and the accompanying sleep disturbances and cognitive impairments^15,54,55^. Therefore, a reduction of SW amplitude may contribute to the observed performance decrements during spaceflights^3,6,12,41,42,56,57^. Given that SWs are only minimally influenced by circadian processes^47,58^, changes observed in this study are likely unrelated to previously discovered circadian misalignments during space missions^4,5,14^.

Ground-based studies attributed a sleep-protective function to sleep spindles^18–22^ that might be compromised during spaceflight. Especially overall sleep spindle density has been shown to correlate with increased sleep stability^19–22^; however, no changes in overall sleep spindle density were detected in the present study. In contrast, an increased occurrence rate of fast spindles during inflight compared to preflight was observed. Postflight fast spindle density did not significantly deviate from inflight or preflight; however, it was between inflight and preflight levels, possibly indicating a renormalization after return to Earth. Our finding of increased fast spindle density could be related to decreased SW amplitudes due to the well-established inverse relationship between SW activity and fast spindle density^59,60^. Notably, no such relationship has been shown for slow spindles^60^, leaving them unaffected, consistent with our results. Alternatively, increased fast spindle density could be related to learning new motor skills while adapting to weightlessness. Support for this hypothesis can be found in several ground-based studies showing that fast spindle density increased after motor sequence learning^32–34^ or visuomotor learning^35,36^. It follows that the fast spindle density should go back to a baseline level once the motor system has adapted to weightlessness; this hypothesis could be tested in long-duration space missions. Unlike SWs, spindles are known to be modulated by circadian processes^58,61,62^. Specifically, spindle density reaches its maximum if the sleep episode is aligned with the circadian rhythm^62^. However, the present results suggest that there was no effect on overall spindle density. Circadian modulation of slow and fast spindle density remains poorly understood.

Slow spindles were shifted to higher frequencies inflight compared to pre- and postflight independent of the sleep stage, potentially reflecting acute effects of the space environment. A similar frequency shift was found for overall sleep spindles inflight compared to preflight, presumably driven by the changes observed in slow spindles. Causes of such shifts in sleep spindle frequency (slow, fast, and overall spindles) remain largely unknown; however, some evidence suggests a role of spindle frequency changes in sleep deficiencies, e.g., sleep spindle frequency is known to increase with age^17,63,64^ while sleep disturbances become more common^65,66^. Spindle frequency is also known to vary with circadian phase^62,67,68^ and might therefore be modulated if sleep schedules are misaligned with circadian rhythmicity as may occur during spaceflight^3,4^. Interestingly, Cheron et al.^69^ have found an increase of the alpha peak frequency (Earth vs. Inflight: 9.9–10.5 Hz), which resides in a similar range as reported here for slow spindles, in five awake ISS crew members when their eyes were closed. Further studies are needed to investigate if there are common neural network properties that could affect both oscillations’ frequencies during spaceflight.

Notably, reduced SW amplitude, reduced sleep duration, and increased spindle frequency have been associated with reduced sleep ability and cognitive impairments in older adults on Earth^15^. Previous studies have related these changes to gray matter thinning^15,54,70,71^ and ß-amyloid accumulation^72,73^. Interestingly, volumetric gray matter decreases were also found in astronauts after return to Earth^74^. Additionally, one study found ß-amyloid accumulation along with cognitive performance decrements in mice after exposing them to cosmic radiation^75^. Future studies are needed to address a possible relationship of sleep disturbances with changes in cortical gray matter and ß-amyloid accumulation.

This study was limited by a small sample size of four astronauts. Because the sample size primarily limits the statistical power to detect a true effect, the present study might have missed other changes related to spaceflight. The small sample size is not expected to drastically inflate false positives because robust statistical methods were used, and residuals were consistent with normality in all models. Furthermore, we conducted robustness checks using reduced LMMs that only included the Condition factor for sleep spindles, slow waves, SPT and TST. These reduced models differed to the full a priori LMM with respect to fast spindle density and duration (see Supplementary Tables 9 and 10). Using the Akaike Information Criterion, we established that our full LMMs are more reliable than the reduced models. In addition, this analysis was limited by the number and placement of the available EEG electrodes. It is known that changes of SW characteristics are pronounced in the frontal lobe^54^, while only central and occipital electrodes were available for the present study. Increases of the effect size of the SW amplitude would be expected if frontal electrodes were analyzed. Moreover, slow and fast spindles follow a topographically distinct distribution, slow spindle activity is expressed more frontally while fast spindle activity predominates central and parietal electrodes^76^. The availability of only central and occipital electrodes might have obscured or weakened effects observed in slow spindles. Additionally, there is no consensus on the frequency ranges of slow and fast spindles and thus between studies overlapping frequency bands are common^76^. Our respective ranges were inspired by more recent studies with a slow spindle range that extends to lower frequencies than traditionally specified^17,76^. Moreover, sleep spindles, as well as slow waves, are influenced by the exogenous administration of melatonin^77–79^. Because of the administration of melatonin in the original experiment, we controlled for possible effects on our measures of interest. Controlling for melatonin in our statistical models should have precluded corruption of our primary results and conclusions. Significant effects of melatonin on slow spindle density and overall spindle amplitude are presented in our Supplementary Tables 12 and 22. Lastly, differing workloads and schedules between conditions and subjects could have affected the outcomes of our analyses^1^.

Our study provides insights on characteristics of neural oscillations during sleep in space, demonstrating fast spindle density and slow spindle frequency increases as well as slow wave amplitude decreases along with reductions of SPT and TST. Our findings suggest reduced sleep ability resulting in lighter as well as shorter sleep during spaceflight relative to Earth consistent with previously reported sleep deficiencies in space. Because insufficient sleep causes a multitude of health and cognitive impairments, it is paramount to understand the underlying mechanisms of sleep deficiency observed during human spaceflight to ensure future missions’ success.

## Methods

### Subjects and study design

Four astronauts (*n* = 4) consented to the analysis of their data which was acquired during two Space Shuttle missions. Written informed consent was obtained prior to their participation. This study was approved by the European Space Agency Medical Review Board (ESA-MB) and the National Aeronautics and Space Administration Johnson Space Center Institutional Review Board (JSC-IRB). We have complied with all relevant ethical regulations.

All night EEG recordings included two baseline measures taken around 30 days before the launch (L-30; preflight-PRE), four measures collected during spaceflight (inflight - IN) of which two were acquired between days 3 and 6 and two between days 12 and 15, and three measures collected within 5 days after return to Earth (postflight-POST). In addition, three all-night recordings were obtained around 7 days before the launch (L-7; preflight-PRE) in three out of the four subjects. One inflight recording session for one subject had to be excluded because of data acquisition issues.

During preflight L-30 and all inflight sessions, three out of four subjects were administered 0.3 mg of melatonin or placebo 30 min before scheduled bedtime on alternate nights to examine the potential effects of the chronobiotic drug on sleep improvement. The present analysis was not interested in the effects of melatonin, however, including the administration of the hypnotic drug as a covariate to account for additional variability in the data was deemed important. Further details about the study design and procedure can be found in Dijk et al.^3^.

### Data acquisition

All night recordings were collected with a modified Vitaport-2 DSR (Temec Instruments, Kerkrade, The Netherlands) using a 12-bit analog-to-digital converter. EEG electrodes were placed according to the International 10–20 System. Dijk et al.^3^ recorded the following electrodes: four EEG electrodes divided onto central (C3, C4) and occipital sites (O1, O2) referenced to the contralateral mastoids (A1, A2), two electrooculogram (EOG; measuring eye movements) electrodes placed next to the left and right outer canthus respectively, and four chin electromyogram (EMG; measuring muscle movements) electrodes distributed across the left and right submental area. EEG signals were sampled at 256 Hz, while EOG and EMG signals were sampled at 128 or 256 Hz. Further details about the data acquisition were published previously^3^.

### Preprocessing

Prior to analyses, the EEG signals were low-pass filtered at 70 Hz, high-pass filtered at 0.5 Hz, and downsampled to 128 Hz. EOG signals were low-pass filtered at 35 Hz, high-pass filtered at 0.16 Hz, and preprocessed with a moving average filter before downsampling to 64 Hz. EMG signals were low-pass filtered at 100 Hz, high-pass filtered at 10.61 Hz, and downsampled to 128 Hz.

Filtered recordings were manually sleep scored according to the AASM rules^80^ using the Python-based scoring software Wonambi (Giovanni Piantoni and Jordan O’Byrne, WONAMBI v5.71 (2019). GitHub repository, https://github.com/wonambi-python/wonambi). Channel sections containing artefacts were visually identified, labeled, and removed before analyses. One inflight recording of a subject had been cut-off or canceled after only one hour due to data acquisition issues. Its short length relative to the other sessions would have confounded the actual spindle and SW estimates, hence this session was excluded from the analyses.

General data preprocessing, visualization and handling were conducted with custom Python scripts using the package MNE^81^.

### Spindle detection

Accumulating evidence suggests that slow and fast spindles are two distinct oscillations with different generating mechanisms and functional roles^17,27–29,82^. For this reason, the present analysis treats slow and fast spindles separately, however, to facilitate comparison with more traditional approaches, overall spindle statistics were also investigated.

Discrete slow and fast spindles were detected using the Python package YASA (Raphael Vallat, YASA v0.1.5 (2019). Zenodo, 10.5281/zenodo.3368609) which is largely based on the algorithm by Lacourse et al.^83^. Fast and slow spindle detection criteria were based on a priori defined frequency ranges generally associated with slow (9–12 Hz) and fast (12–15 Hz) spindles^27^ (Fig. 1a). The algorithm uses a 300 ms window with a step size of 100 ms to compute the moving root mean squared (RMS) of the filtered EEG data within the defined slow spindle and fast spindle frequency bands. The same sliding window and step size values are then used to compute a moving correlation between the broadband signal (0.5–40 Hz) and the EEG signal filtered in the respective spindle band. Next, the relative power in the spindle band with respect to the total power (0.5–40 Hz) was estimated based on Short-Time Fourier Transforms with 2 s windows and a 200 ms overlap. Sleep spindles are identified if the moving RMS crosses the RMS_mean_ + 1.5 RMS_SD_ threshold (RMS mean and standard deviation were computed from the filtered EEG), or if the moving correlation is above 0.65, or if the relative power exceeds the relative power threshold that was determined for each subject and stage (N2 or N3) based on a placebo night during L-30 (average relative power across all channels). A spindle was detected when two out of the three thresholds were simultaneously reached. Detected spindles shorter than 0.5 s or longer than 2 s were discarded. Spindles occurring in different channels within 500 ms of each other were assumed to reflect the same spindle. In these cases, the spindle with the maximum relative power was selected for further analysis.

To analyze overall spindle statistics, discrete oscillations were detected using the same approach as outlined for slow and fast spindles with an a priori defined frequency range from 9 to 15 Hz (Fig. 1a), which is in alignment with more traditional analyses^20,27,84^.

Next, sleep spindle densities (number of sleep spindles per minute) as well as discrete sleep spindles’ peak-to-peak amplitudes, frequencies, and durations for each subject, session, condition, stage, and channel were obtained.

### Slow wave detection

SWs were detected in the left central channel C3-A2 of stage N3. We excluded N2 epochs from the analysis to avoid contamination of the detected slow waves with K-complexes that are characteristic of N2 sleep^85–88^. We used YASA’s slow-wave detector, which is based on algorithms by Massimini et al.^89^ and Carrier et al.^54^. The algorithm was set to identify discrete SWs with a frequency of 0.5–3.5 Hz (Fig. 1a) using a linear phase Finite Impulse Response filter with a 0.2 Hz transition band. Within the determined bandpass frequencies SWs of negative trough amplitudes > 40 µV and < 300 µV, and positive peak amplitudes > 10 µV and < 200 µV were detected. After sorting identified negative peaks with subsequent positive peaks, the algorithm computes peak-to-peak amplitudes and retains SWs between 75 and 500 µV. Finally, SW down-states lasting > 300 and < 1500 ms, and up-states lasting > 100 ms and < 1000 ms are retained for further analysis. Afterwards, SW densities (number of SWs per minute), discrete SWs’ peak-to-peak amplitudes, durations, and slopes were obtained for each subject, session, and condition in channel C3-A2.

### Spectral band-power estimation

Sleep spindle and SW spectral band-power were estimated for N2 (spindles only) and N3 (spindles and SWs) by integrating the power spectral density (PSD) within the frequency band of interest (slow spindles: 9–12 Hz, fast spindles: 12–15 Hz, overall spindles: 9–15 Hz, SWs: 0.5–3.5 Hz; Fig. 1a). To this end, the PSD was estimated using Welch’s method^90^ with 8 s data-segments that had a 50% overlap to which a Hanning window was applied. PSD estimates were obtained for all subjects and conditions. In the case of sleep spindles PSD estimates were averaged across channels while for SWs only channel C3-A2 was used.

### Statistical analysis

Statistical analyses of sleep period time (SPT; time spent asleep plus wake after sleep onset), total sleep time (TST; time spent asleep minus wake after sleep onset), time spent in each sleep stage, time spent in wake after sleep onset (WASO) as well as sleep spindle and SW characteristics were conducted with linear mixed-effects models (LMM) as implemented in the R (R Core Team, 2019) package afex^91^. LMMs were chosen for this analysis to account for the within-subjects design and for unbalanced data resulting from melatonin not being administered to one of the subjects and the remaining subjects not receiving melatonin postflight. Melatonin (active, placebo) was included as a covariate (non-interacting fixed effect) in all models to account for additional variability in the data but was not of primary interest. Fixed effects were included according to a priori hypotheses and hence no model selection was done. A random effect with unique subject identifiers (SubjectID) as a grouping factor was included in all models to account for the dependence of within-subjects measures.

Global sleep statistics were analyzed by fitting a LMM to SPT and TST with a fixed effect for Condition (PRE, IN, POST). To investigate whether the duration of individual sleep stages including WASO are affected by spaceflight, another LMM was fitted with the fixed effects Condition (PRE, IN, POST) and Stage (WASO, N1, N2, N3, REM), as well as their interaction Condition × Stage.

The characteristics of detected sleep spindles were averaged within sleep stages (N2, N3), melatonin intake (active, placebo), condition (PRE, IN, POST), and subjects. Data were then fitted with a LMM including the fixed effects Condition (PRE, IN, POST) and Stage (N2, N3), as well as their interaction Condition × Stage.

Similarly, slow wave characteristics were averaged within the conditions (PRE, IN, POST), melatonin intake (active, placebo), and subjects. Next, the data were fitted with LMMs including a fixed effect for Condition (PRE, IN, POST).

All models were fitted by restricted maximum likelihood. After fitting LMMs the residuals were tested for consistency with the normal distribution using Shapiro—Wilk tests. None of the LMMs presented in this study violated the normality assumption. Hypothesis testing on the fixed effects was done with F-tests (Type III sums of squares) using the Kenward—Roger approximation for degrees of freedom. Post hoc two-sided Tukey HSD tests were performed on the estimated marginal means (EMMs) to control the family-wise error rate using the R package emmeans (Russell Lenth (2019). emmeans: EMMs, aka Least-Squares Means. R package version 1.4. https://CRAN.R-project.org/package=emmeans). Unless stated otherwise, unstandardized effect sizes based on the differences between EMMs along with their standard error are reported^92^. Post hoc results of significant Stage or Melatonin main effects are reported in the supplementary information because they were not of primary interest in this study and do not affect conclusions based on Condition main effects or Condition × Stage interactions.

We conducted parallel analyses for sleep spindles, slow waves, SPT, and TST using reduced LMMs that only included the Condition factor to reduce the danger of overfitting given the limited sample size (*n* = 4) and to ensure the robustness of our a priori defined full LMM results (see Supplementary Tables for reduced models). These control analyses deviated only for fast spindle density and duration. Notably, for both fast spindle measures the Akaike Information Criterion, a measure that considers the model complexity and goodness-of-fit, corroborated that our a priori defined full LMMs performed better (see Supplementary Tables 9 and 10).

### Reporting summary

Further information on research design is available in the Nature Research Reporting Summary linked to this article.

## Supplementary information

Supplementary Information

Reporting Summary

## Data Availability

The data supporting the findings of this study are available on request from the National Aeronautics and Space Administration’s Life Sciences Data Archive. The data are not publicly available due to privacy restrictions.

## Code availability

All relevant scripts will be made available from the corresponding authors upon request.
