# Supplementary material for: Altered sleep spindles and slow waves during space shuttle missions
Source: NPJ Microgravity. 2021 Nov 18;7:48. doi: 10.1038/s41526-021-00177-1 (PMC8602337; doi:10.1038/s41526-021-00177-1)
Supplement: Supplementary file 1 — Supplementary Information [file 41526_2021_177_MOESM1_ESM.pdf]

Supplementary Information

Supplementary Results

Sleep Statistics

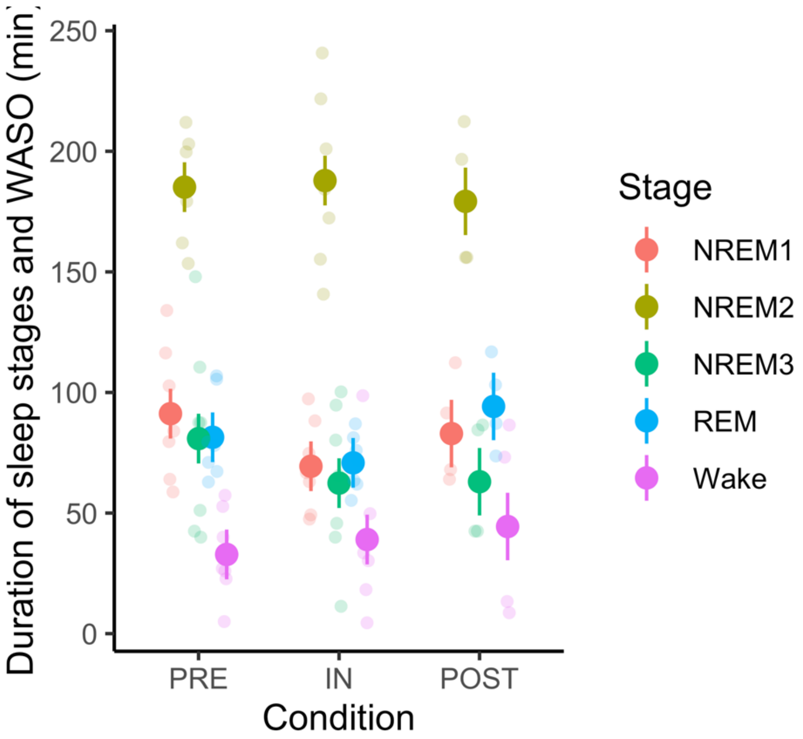

Supplementary Figure 1. Duration of sleep stages and WASO. The large dots represent the estimated marginal means (± standard error) of each condition separated by sleep stages or WASO (Wake). The smaller dots in the background are individual data points.

Fast Spindles – Stage and Melatonin Contrasts

Stage	Mean (1/min)	SE	df	$CI_{95\% \, lower}$	$CI_{95\% \, higher}$		
N2	7.531	2.288	3.089	0.368	14.693		
N3	3.500	2.288	3.089	-3.663	10.662		
**Contrast**	**Effect Size (1/min)**	**SE**	**df**	**t**	**p**	**$CI_{95\% \, lower}$**	**$CI_{95\% \, higher}$**
N2 - N3	4.031	0.482	26.000	8.365	<.001	3.041	5.021

Supplementary Table 1. Fast spindle density post-hoc Stage EMMs and contrasts.

Stage	Mean (µV)	SE	df	$CI_{95\% \, lower}$	$CI_{95\% \, higher}$		
N2	41.455	5.438	3.027	24.238	58.673		
N3	44.131	5.438	3.027	26.913	61.348		
**Contrast**	**Effect Size (µV)**	**SE**	**df**	**t**	**p**	**$CI_{95\% \, lower}$**	**$CI_{95\% \, higher}$**
N2 - N3	-2.675	0.636	26.000	-4.207	0.000	-3.982	-1.368

Supplementary Table 2. Fast spindle amplitude post-hoc Stage EMMs and contrasts.

Stage	Mean (μV^2)	SE	df	$CI_{95\% \, lower}$	$CI_{95\% \, higher}$		
N2	5.670	1.686	3.027	0.331	11.009		
N3	5.117	1.686	3.027	-0.222	10.456		
**Contrast**	**Effect Size (μV^2)**	**SE**	**df**	**t**	**p**	**$CI_{95\% \, lower}$**	**$CI_{95\% \, higher}$**
N2 - N3	0.553	0.197	26	2.812	0.009	0.149	0.957

Supplementary Table 3. Fast spindle bandpower post-hoc Stage EMMs and contrasts.

Stage	Mean (s)	SE	df	$CI_{95\% \, lower}$	$CI_{95\% \, higher}$		
N2	0.828	0.040	3.047	0.703	0.953		
N3	0.756	0.040	3.047	0.631	0.881		
**Contrast**	**Effect Size (s)**	**SE**	**df**	**t**	**p**	**$CI_{95\% \, lower}$**	**$CI_{95\% \, higher}$**
N2 - N3	0.072	0.006	26.000	11.900	0.000	0.060	0.085

Supplementary Table 4. Fast spindle duration post-hoc Stage EMMs and contrasts.

Stage	Mean (Hz)	SE	df	$CI_{95\% \, lower}$	$CI_{95\% \, higher}$
N2	13.292	0.080	3.325	13.051	13.533

Stage	Mean (Hz)	SE	df	$CI_{95\% lower}$	$CI_{95\% higher}$
N3	13.193	0.080	3.325	12.952	13.434

Contrast	Effect Size (Hz)	SE	df	t	p	$CI_{95\% lower}$	$CI_{95\% higher}$
N2 - N3	0.099	0.031	26.000	3.146	0.004	0.034	0.164

Supplementary Table 5. Fast spindle frequency post-hoc Stage EMMs and contrasts.

Fast Spindles – Reduced Linear Mixed Models

Effect	df	F	p
Condition	2, 30.00	2.89	.07

Supplementary Table 6. Fast spindle bandpower reduced LMM results.

Effect	df	F	p
Condition	2, 30.00	3.11	.06

Supplementary Table 7. Fast spindle amplitude reduced LMM results.

Effect	df	F	p
Condition	2, 30.03	2.22	.13

Supplementary Table 8. Fast spindle frequency reduced LMM results.

Effect	df	F	p
Condition	2, 30.02	1.72	.20
**Model**	**AIC**		
Full	155.565		
Reduced	187.108		

Supplementary Table 9. Fast spindle density reduced LMM results and Model comparison between the full and reduced LMMs using the Akaike Information Criterion (AIC).

Effect	df	F	p				
Condition	2, 6	10.37 *	.01				
**Condition**	**Mean (s)**	**SE**	**df**	**$CI_{95\% lower}$**	**$CI_{95\% higher}$**		
in	0.831	0.037	3.046	0.713	0.949		
post	0.818	0.037	3.046	0.701	0.936		
pre	0.806	0.037	3.046	0.688	0.923		
**Contrast**	**Effect Size (s)**	**SE**	**df**	**t**	**p**	**$CI_{95\% lower}$**	**$CI_{95\% higher}$**

Effect	df	F	p				
in - post	0.013	0.006	6.000	2.282	0.135	-0.004	0.030
in - pre	0.026	0.006	6.000	4.554	0.009	0.008	0.043
post - pre	0.013	0.006	6.000	2.272	0.137	-0.004	0.030

**Model**	**AIC**
**Full**	-96.159
**Reduced**	-28.461

Supplementary Table 10. Fast spindle duration reduced LMM results and Model comparison between the full and reduced LMMs using the Akaike Information Criterion (AIC). **NOTE**: This reduced model showed no significant effect of Condition on fast spindle duration using the full data, however, the residuals were inconsistent with the normality assumption. To mitigate this issue, we aggregated the data across Stage and Melatonin.

Slow Spindles – Stage and Melatonin Contrasts

Stage	Mean (1/min)	SE	df	$CI_{95\%\ lower}$	$CI_{95\%\ higher}$
N2	9.269	1.971	3.098	3.107	15.430
N3	6.193	1.971	3.098	0.031	12.355

Contrast	Effect Size (1/min)	SE	df	t	p	$CI_{95\%\ lower}$	$CI_{95\%\ higher}$
N2 - N3	3.076	0.434	26.000	7.085	<.001	2.184	3.968

Supplementary Table 11. Slow spindle density post-hoc Stage EMMs and contrasts.

Melatonin	Mean (1/min)	SE	df	$CI_{95\%\ lower}$	$CI_{95\%\ higher}$
active	8.413	1.990	3.220	2.317	14.509
placebo	7.048	1.960	3.028	0.845	13.252

Contrast	Effect Size (1/min)	SE	df	t	p	$CI_{95\%\ lower}$	$CI_{95\%\ higher}$
active - placebo	1.365	0.503	26.077	2.713	0.012	0.331	2.399

Supplementary Table 12. Slow spindle density post-hoc Melatonin EMMs and contrasts.

Stage	Mean (µV)	SE	df	$CI_{95\%\ lower}$	$CI_{95\%\ higher}$
N2	43.839	5.365	3.042	26.898	60.779
N3	47.278	5.365	3.042	30.337	64.219

Contrast	Effect Size (µV)	SE	df	t	p	$CI_{95\%\ lower}$	$CI_{95\%\ higher}$
N2 - N3	-3.439	0.780	26.000	-4.408	<.001	-5.043	-1.836

Supplementary Table 13. Slow spindle amplitude post-hoc Stage EMMs and contrasts

Stage	Mean (s)	SE	df	$CI_{95\%\ lower}$	$CI_{95\%\ higher}$
N2	0.883	0.026	3.124	0.803	0.962
N3	0.833	0.026	3.124	0.754	0.913

Contrast	Effect Size (s)	SE	df	t	p	$CI_{95\%\ lower}$	$CI_{95\%\ higher}$
N2 - N3	0.049	0.006	26.000	7.792	<.001	0.036	0.062

Supplementary Table 14. Slow spindle duration post-hoc Stage EMMs and contrasts

Slow Spindles – Reduced Linear Mixed Models

Effect	df	F	p
Condition	2, 30.01	0.38	.69

Supplementary Table 15. Slow spindle bandpower reduced LMM results.

Effect	df	F	p
Condition	2, 30.01	1.27	.29

Supplementary Table 16. Slow spindle amplitude reduced LMM results.

Effect	df	F	p
Condition	2, 30.02	0.99	.38

Supplementary Table 17. Slow spindle density reduced LMM results.

Effect	df	F	p
Condition	2, 30.02	0.21	.81

Supplementary Table 18. Slow spindle duration reduced LMM results.

Effect	df	F	p
Condition	2, 30.01	19.26 ***	<.0001

Condition	Mean (Hz)	SE	df	$CI_{95\% \text{ lower}}$	$CI_{95\% \text{ higher}}$
in	10.239	0.128	3.095	9.838	10.640
post	10.097	0.129	3.208	9.700	10.494
pre	10.069	0.128	3.095	9.667	10.470

Contrast	Effect Size (Hz)	SE	df	t	p	$CI_{95\% \text{ lower}}$	$CI_{95\% \text{ higher}}$
in - post	0.142	0.034	30.009	4.183	0.001	0.058	0.225
in - pre	0.170	0.029	30.000	5.926	0.000	0.099	0.241
post - pre	0.028	0.034	30.009	0.841	0.681	-0.055	0.112

Supplementary Table 19. Slow spindle frequency reduced LMM results.

Overall Spindles – Stage and Melatonin Contrasts

Stage	Mean (1/min)	SE	df	$CI_{95\% lower}$	$CI_{95\% higher}$
N2	12.853	1.818	3.588	7.567	18.139
N3	3.047	1.818	3.588	-2.239	8.333

Contrast	Effect Size (1/min)	SE	df	t	p	$CI_{95\% lower}$	$CI_{95\% higher}$
N2 - N3	9.806	0.935	26.001	10.484	<.001	7.883	11.728

Supplementary Table 20. Overall spindle density post-hoc Stage EMMs and contrasts.

Stage	Mean (µV)	SE	df	$CI_{95\% lower}$	$CI_{95\% higher}$
N2	44.900	5.278	3.043	28.235	61.564
N3	48.326	5.278	3.043	31.662	64.990

Contrast	Effect Size (µV)	SE	df	t	p	$CI_{95\% lower}$	$CI_{95\% higher}$
N2 - N3	-3.426	0.773	26.000	-4.433	<.001	-5.015	-1.837

Supplementary Table 21. Overall spindle amplitude post-hoc Stage EMMs and contrasts.

Melatonin	Mean (µV)	SE	df	$CI_{95\% lower}$	$CI_{95\% higher}$
active	45.599	5.301	3.096	29.020	62.177
placebo	47.627	5.265	3.012	30.911	64.343

Contrast	Effect Size (µV)	SE	df	t	p	$CI_{95\% lower}$	$CI_{95\% higher}$
active - placebo	-2.028	0.895	26.034	-2.265	0.032	-3.869	-0.188

Supplementary Table 22. Overall spindle amplitude post-hoc Melatonin EMMs and contrasts.

Stage	Mean (s)	SE	df	$CI_{95\% lower}$	$CI_{95\% higher}$
N2	0.921	0.026	3.124	0.839	1.003
N3	0.858	0.026	3.124	0.776	0.940

Contrast	Effect Size (s)	SE	df	t	p	$CI_{95\% lower}$	$CI_{95\% higher}$
N2 - N3	0.063	0.007	26.000	9.571	<.001	0.049	0.076

Supplementary Table 23. Overall spindle duration post-hoc Stage EMMs and contrasts.

Stage	Mean (Hz)	SE	df	$CI_{95\%\ lower}$	$CI_{95\%\ higher}$
N2	11.431	0.347	3.045	10.336	12.526
N3	11.277	0.347	3.045	10.181	12.372

Contrast	Effect Size (Hz)	SE	df	t	p	$CI_{95\%\ lower}$	$CI_{95\%\ higher}$
N2 – N3	0.154	0.052	26.000	2.953	0.007	0.047	0.262

Supplementary Table 24. Overall spindle frequency post-hoc Stage EMMs and contrasts.

Overall Spindles – Reduced Linear Mixed Models

Effect	df	F	p
Condition	2, 30.00	1.96	.16

Supplementary Table 25. Overall spindle bandpower reduced LMM results.

Effect	df	F	p
Condition	2, 30.01	1.43	.25

Supplementary Table 26. Overall spindle amplitude reduced LMM results.

Effect	df	F	p
Condition	2, 6	0.28	.76

Supplementary Table 27. Overall spindle density. **NOTE**: This reduced model showed no significant effect of Condition on overall spindle density using the full data, however, the residuals were inconsistent with the normality assumption. To mitigate this issue, we aggregated the data across Stage and Melatonin.

Effect	df	F	p
Condition	2, 6	0.52	.62

Supplementary Table 28. Overall spindle duration reduced LMM results. **NOTE**: This reduced model showed no significant effect of Condition on overall spindle duration using the full data, however, the residuals were inconsistent with the normality assumption. To mitigate this issue, we aggregated the data across Stage and Melatonin.

Effect	df	F	p
Condition	2, 30.00	8.18 **	.001

Condition	Mean (Hz)	SE	df	$CI_{95\%\ lower}$	$CI_{95\%}$
in	11.500	0.343	3.071	10.422	12.578
post	11.404	0.345	3.154	10.335	12.474
pre	11.234	0.343	3.071	10.156	12.312

Contrast	Effect Size (Hz)	SE	df	t	p	$CI_{95\%\ lower}$	$CI_{95\%\ higher}$
in - post	0.096	0.078	30.007	1.226	0.447	-0.097	0.289
in - pre	0.266	0.066	30.000	4.012	0.001	0.103	0.430
post - pre	0.170	0.078	30.007	2.175	0.092	-0.023	0.363

Supplementary Table 29. Overall spindle frequency reduced LMM results.

Overall Sleep Statistics

Effect	df	F	p
Condition	2, 12.22	15.73	.0004

Condition	Mean (min)	SE	df	$CI_{95\%\ lower}$	$CI_{95\%\ higher}$
in	430.238	5.767	11.640	417.629	442.847
post	468.833	7.522	14.940	452.795	484.872
pre	472.214	5.767	11.640	459.605	484.824

Contrast	Effect Size (min)	SE	df	t	p	$CI_{95\%\ lower}$	$CI_{95\%\ higher}$
in - post	-38.595	9.479	12.302	-4.072	0.004	-63.800	-13.391
in - pre	-41.976	8.042	12.097	-5.220	0.001	-63.407	-20.545
post - pre	-3.381	9.479	12.302	-0.357	0.933	-28.586	21.824

Supplementary Table 30. Sleep period time reduced LMM results.

Effect	df	F	p
Condition	2, 12.11	5.02	.03

Condition	Mean (min)	SE	df	$CI_{95\%\ lower}$	$CI_{95\%\ higher}$
in	389.433	13.191	7.864	358.923	419.943
post	423.417	16.107	12.549	388.492	458.342
pre	437.593	13.191	7.864	407.084	468.103

Contrast	Effect Size (min)	SE	df	t	p	$CI_{95\%\ lower}$	$CI_{95\%\ higher}$
in - post	-33.984	18.271	12.164	-1.860	0.192	-82.640	14.672
in - pre	-48.161	15.478	12.028	-3.111	0.023	-89.442	-6.879
post - pre	-14.177	18.271	12.164	-0.776	0.724	-62.833	34.479

Supplementary Table 31. Total sleep time reduced LMM results.
