# Supplementary material for: Altered sleep spindles and slow waves during space shuttle missions
Source: NPJ Microgravity. 2021 Nov 18;7:48. doi: 10.1038/s41526-021-00177-1 (PMC8602337; doi:10.1038/s41526-021-00177-1)
Supplement: Supplementary file 2 — Reporting Summary [file 41526_2021_177_MOESM2_ESM.pdf]

nature portfolio

Corresponding author(s): Dominik Koller

Last updated by author(s): Aug 1, 2021

Reporting Summary

Nature Portfolio wishes to improve the reproducibility of the work that we publish. This form provides structure for consistency and transparency in reporting. For further information on Nature Portfolio policies, see our Editorial Policies and the Editorial Policy Checklist.

Statistics

For all statistical analyses, confirm that the following items are present in the figure legend, table legend, main text, or Methods section.

n/a	Confirmed	
☐	☒	The exact sample size (n) for each experimental group/condition, given as a discrete number and unit of measurement
☐	☒	A statement on whether measurements were taken from distinct samples or whether the same sample was measured repeatedly
☐	☒	The statistical test(s) used AND whether they are one- or two-sided<br>*Only common tests should be described solely by name; describe more complex techniques in the Methods section.*
☐	☒	A description of all covariates tested
☐	☒	A description of any assumptions or corrections, such as tests of normality and adjustment for multiple comparisons
☐	☒	A full description of the statistical parameters including central tendency (e.g. means) or other basic estimates (e.g. regression coefficient) AND variation (e.g. standard deviation) or associated estimates of uncertainty (e.g. confidence intervals)
☐	☒	For null hypothesis testing, the test statistic (e.g. F, t, r) with confidence intervals, effect sizes, degrees of freedom and P value noted<br>*Give P values as exact values whenever suitable.*
☒	☐	For Bayesian analysis, information on the choice of priors and Markov chain Monte Carlo settings
☒	☐	For hierarchical and complex designs, identification of the appropriate level for tests and full reporting of outcomes
☐	☒	Estimates of effect sizes (e.g. Cohen's d, Pearson's r), indicating how they were calculated

Our web collection on statistics for biologists contains articles on many of the points above.

Software and code

Policy information about availability of computer code

Data collection	No software was used for data collection. We re-analyzed data that was previously recorded by Dijk, D. J. et al. Sleep, performance, circadian rhythms, and light-dark cycles during two space shuttle flights. Am. J. Physiol. Regul. Integr. Comp. Physiol. 281, R1647-64 (2001).
Data analysis	We made use of the standard free open source frameworks Python and R. Specific Python and R package versions are specified in our manuscript.

For manuscripts utilizing custom algorithms or software that are central to the research but not yet described in published literature, software must be made available to editors and reviewers. We strongly encourage code deposition in a community repository (e.g. GitHub). See the Nature Portfolio guidelines for submitting code & software for further information.

Data

Policy information about availability of data

All manuscripts must include a data availability statement. This statement should provide the following information, where applicable:

- Accession codes, unique identifiers, or web links for publicly available datasets
- A description of any restrictions on data availability
- For clinical datasets or third party data, please ensure that the statement adheres to our policy

The data supporting the findings of our study are available on request from the National Aeronautics and Space Administration’s Life Sciences Data Archive. The data are not publicly available due to privacy restrictions.

Field-specific reporting

Please select the one below that is the best fit for your research. If you are not sure, read the appropriate sections before making your selection.

☒ Life sciences ☐ Behavioural & social sciences ☐ Ecological, evolutionary & environmental sciences

For a reference copy of the document with all sections, see nature.com/documents/nr-reporting-summary-flat.pdf

Life sciences study design

All studies must disclose on these points even when the disclosure is negative.

Sample size	Our study includes very rare sleep EEG recordings from astronauts that took part in two space shuttle missions. Our sample size was limited by the retrospective nature of our study. Nevertheless, we calculated possible effect sizes for our primary hypotheses given our sample size and concluded that the sample size was sufficient to detect meaningful effect sizes reported in previous ground-based literature.
Data exclusions	One inflight recording of a subject had been cut-off or cancelled after only one hour due to data acquisition issues. Its short length relative to the other sessions would have confounded the actual spindle and slow wave estimates, hence this session was excluded from the analyses.
Replication	The analyzed data are very rare sleep EEG recordings from astronauts that took part in two space shuttle missions. Due to the nature of these recordings our findings are original and require the acquisition of new sleep EEG data during human spaceflight for further replication. However, to ensure replicability, all our analyses were conducted with the freely available open source software frameworks Python and R.
Randomization	Our study uses a within-subjects design and therefore all subjects participated in every experimental condition (pre-, in-, and post-flight).
Blinding	The recording session within experimental conditions were unbalanced and could easily be identified by the investigators. Therefore, investigators could not be blind with respect to the experimental condition.

Reporting for specific materials, systems and methods

We require information from authors about some types of materials, experimental systems and methods used in many studies. Here, indicate whether each material, system or method listed is relevant to your study. If you are not sure if a list item applies to your research, read the appropriate section before selecting a response.

Materials & experimental systems

n/a	Involved in the study	
☒	☐	Antibodies
☒	☐	Eukaryotic cell lines
☒	☐	Palaeontology and archaeology
☒	☐	Animals and other organisms
☐	☒	Human research participants
☒	☐	Clinical data
☒	☐	Dual use research of concern

Methods

n/a	Involved in the study	
☒	☐	ChIP-seq
☒	☐	Flow cytometry
☒	☐	MRI-based neuroimaging

Human research participants

Policy information about studies involving human research participants

Population characteristics	Four astronauts consented to participating in our study. To ensure the privacy of the small pool of astronauts that participated in our study NASA's Life Sciences Data Archive and the Lifetime Surveillance of Astronaut Health advisory board prohibited disclosure of astronaut demographics within our manuscript. Notably, we did not draw any conclusions based on sex or age in our manuscript.
Recruitment	The astronauts that participated in the original study by Dijk et al (2001) were requested to participate in our retrospective analysis by NASA's Life Sciences Data Archive. Our study team was not directly involved in recruiting the participants for our study.
Ethics oversight	European Space Agency Medical Board and National Aeronautics and Space Administration Institutional Review Board

Note that full information on the approval of the study protocol must also be provided in the manuscript.
